# FLGR: Fixed Length Gists Representation Learning for RNN-HMM Hybrid-Based Neuromorphic Continuous Gesture Recognition

**DOI:** 10.3389/fnins.2019.00073

**Published:** 2019-02-12

**Authors:** Guang Chen, Jieneng Chen, Marten Lienen, Jörg Conradt, Florian Röhrbein, Alois C. Knoll

**Affiliations:** ^1^College of Automotive Engineering, Tongji University, Shanghai, China; ^2^Chair of Robotics, Artificial Intelligence and Real-time Systems, Technische Universität München, Munich, Germany; ^3^College of Electronics and Information Engineering, Tongji University, Shanghai, China; ^4^Department of Computational Science and Technology, KTH Royal Institute of Technology, Stockholm, Sweden

**Keywords:** representation learning, neuromorphic vision, continuous gesture recognition, mixture density autoencoder, recurrent neural network, hidden markov model

## Abstract

A neuromorphic vision sensors is a novel passive sensing modality and frameless sensors with several advantages over conventional cameras. Frame-based cameras have an average frame-rate of 30 fps, causing motion blur when capturing fast motion, e.g., hand gesture. Rather than wastefully sending entire images at a fixed frame rate, neuromorphic vision sensors only transmit the local pixel-level changes induced by the movement in a scene when they occur. This leads to advantageous characteristics, including low energy consumption, high dynamic range, a sparse event stream and low response latency. In this study, a novel representation learning method was proposed: Fixed Length Gists Representation (FLGR) learning for event-based gesture recognition. Previous methods accumulate events into video frames in a time duration (e.g., 30 ms) to make the accumulated image-level representation. However, the accumulated-frame-based representation waives the friendly event-driven paradigm of neuromorphic vision sensor. New representation are urgently needed to fill the gap in non-accumulated-frame-based representation and exploit the further capabilities of neuromorphic vision. The proposed FLGR is a sequence learned from mixture density autoencoder and preserves the nature of event-based data better. FLGR has a data format of fixed length, and it is easy to feed to sequence classifier. Moreover, an RNN-HMM hybrid was proposed to address the continuous gesture recognition problem. Recurrent neural network (RNN) was applied for FLGR sequence classification while hidden Markov model (HMM) is employed for localizing the candidate gesture and improving the result in a continuous sequence. A neuromorphic continuous hand gestures dataset (Neuro ConGD Dataset) was developed with 17 hand gestures classes for the community of the neuromorphic research. Hopefully, FLGR can inspire the study on the event-based highly efficient, high-speed, and high-dynamic-range sequence classification tasks.

## 1. Introduction

Gesture recognition has aroused rising attentions because of its emerging significance in many robotic applications e.g., safe human-robot cooperation in an industrial environment. However, conventional camera-based gesture recognition exhibits two major drawbacks. First, the reaction speed of the conventional camera is limited by its frame rate, typically 30 fps, causing motion blur when capturing fast hand motions. Second, the accumulated-frame-based visual acquisition can lead to data redundancy and memory requirement, thereby hampering the large scale commercial usage in embedded systems. Compared with conventional cameras, neuromorphic vision sensors as a bio-inspired sensor do not capture full images at a fixed frame-rate. Besides, they characterized by high temporal resolution (microseconds), high dynamic range (120–140 dB), low power and low bandwidth. Neuromorphic vision represents a paradigm shift in computer vision because of its principle of the operation and the unconventional output.

However, current study on neuromorphic gesture recognition all belongs to segmented gesture recognition. For segmented gesture recognition, the scenario of the problem can be simply described as classifying a well-delineated sequence of video frames as one of a set of gesture types. This is in contrast to continuous/online human gesture recognition where there are no a priori given boundaries of gesture execution (Aggarwal and Ryoo, 2011; Wang et al., 2018). It is meaningful to develop novel architecture for neuromorphic continuous gesture recognition, which is the first step to achieve online recognition.

However, given the events nature of variable length and asynchronous sequence, it is not suitable for feeding the events to common classifier directly for sequence classification tasks e.g., gesture recognition. Existing works accumulate neuromorphic sensor's output events in a duration (e.g., 30 ms), and denote them as image frame (Moeys et al., 2016). These methods perform the classification and recognition task on an image level, thereby waiving the nature of events. Hence, new representations and technologies are urgently needed to exploit the capabilities of neuromorphic vision. The aim of this study was twofold: to explore a novel representation of neuromorphic events and to investigate the ability to translate successes in field of deep learning into neuromorphic vision in gesture recognition.

### 1.1. Neuromorphic Vision Sensor

The dynamic vision sensor (DVS), a type of neuromorphic vision sensor (Lichtsteiner et al., 2008), was employed to acquire the hand gesture data. The design of neuromorphic vision sensors is inspired by the way vision happens on the retina of a biological eye, e.g., the human eye, which is reflected in its eponymous attributes, including asynchronous and temporal contrast. The former indicates that each of the DVS pixels leads to an intensity change once it is triggered as opposed to the synchronous way in which a conventional camera queries all pixels at once every few milliseconds. The latter implied that a pixel is triggered when the variation in light intensity at its position exceeds a certain threshold. These attributes make the pixels of the DVS comparable to retinal ganglion cells.

The DVS applied here has a spatial resolution of 128 × 128 pixels as well as a temporal resolution of microseconds, suggesting that events are timestamped by a free-running counter ticking up at 11 kHz. Each pixel circuit tracks the temporal contrast defined as light log-intensity. An event is triggered every time the temporal contrast passes a threshold θ. The whole process exhibits a latency of 15 μs. The DVS streams events over USB in address-event representation (AER). In AER, each event is a 4-tuple (*t, x, y, p*) where *t* denotes the timestamp; *x* and *y* are the coordinates of the event's origin; *p* is the event's polarity.

### 1.2. Representation for Neuromorphic Vision

Since the stream of neuromorphic events is asynchronous and variable in length, researchers tried to represent them as another type of data easy to process for later detection and recognition tasks. Existing methods for representation of DVS events are divided into 4 types, namely the fully accumulated-frame-based representation, the semi-accumulated-frame-based representation, the reconstructed-frame-based representation and the non-accumulated-frame-based representation. First, the fully accumulated frame-based representation is the most broadly used representation of neuromorphic events. Park et al. (2016) and Maqueda et al. (2018) accumulated the events into the frame with a duration of 30 ms in average. Vidal et al. (2018) collapsed every spatio-temporal window of events to a synthetic accumulated frame by drawing each event on the image frames. They used FAST corner detector to extract features on the frames. Second, the events were processed by the semi-accumulated-frame-based representation before being accumulated into a frame (Lee et al., 2014; Mueggler et al., 2015). Mueggler et al. (2015) processed the events by the lifetime estimation and accumulated them to yield the shape gradient image. Lee et al. (2014) processed the events by means of leaky integrate-and-fire (LIF) neurons and clustered a moving hand by accumulating the output events from LIF with a 3-ms interval. Third, Bardow et al. (2016) and Munda et al. (2018) exploited intensity change to reconstruct the gray image. However, noted that all three methods above process the events on an accumulated image frame level. Since the transformed images are often blurred and redundant, the image-level preprocessing negatively affects model performance and abandons the hardware friendly event-driven paradigm. As a result, such methods waive the the nature of events data and lead to unnecessary redundancy of data and memory requirement. In recent years, the processing of event sequence is no longer on an level of image, but more focused on the natural processing of event sequence (Neil et al., 2016; Wu et al., 2018). Wu et al. (2018) first trained an event-driven LSTM and prove the capability of recurrent neural network (RNN) to process event-based classification task. Note that they applied their framework on N-MNIST dataset, which is a toll dataset of handwritten digits. A review paper (Cadena et al., 2016) highlighted that the main bottleneck of event-based computer vision is how to represent events sequence appropriately. Since the output consists of a sequence of asynchronous events, traditional frame-based computer-vision algorithms are not applicable. This requires a paradigm shift from the traditional computer vision approaches developed over the past 5 decades. They explained that the design goal of such algorithms is to preserve the event-based nature of the sensor. Thus, it is necessary to further prove the capability of the non-accumulated-image-based representation by applying them to event-driven tasks.

### 1.3. Related Works

Under the recent development of deep learning (Krizhevsky et al., 2012), many methods used for hand gesture recognition with conventional cameras have been presented based on Convolutional Neural Networks (ConvNets) (Ji et al., 2013; Neverova et al., 2014; Molchanov et al., 2015; Knoller et al., 2016; Sinha et al., 2016) and RNN (Ohn-Bar and Trivedi, 2014; Neverova et al., 2016; Wu et al., 2016). Among these frameworks, RNNs are attractive because they equip neural networks with memories for temporal tasks, and the introduction of gating units e.g., LSTM and GRU (Hochreiter and Schmidhuber, 1997; Cho et al., 2014) has significantly contributed to making the learning of these networks manageable. In general, deep-learning-based methods outperform traditional handcrafted-feature-based methods in gesture recognition task (Wang et al., 2018).

All the efforts above rely on conventional cameras at fixed frame-rate. Conventional cameras will suffer from various motion-related artifacts (motion blur, rolling shutter, etc.) which may affect the performance for the rapid gesture recognition. In contrast, the event data generated by neuromorphic vision sensors are natural *motion detectors* and automatically filter out any temporally redundant information. The DVS is promising sensor for low latency and low bandwidth tasks. A robotic goal keeper was presented in Delbruck and Lang (2013) with a reaction time of 3 ms. Robot localization was demonstrated by Mueggler et al. (2014) using a DVS during high-speed maneuvers, in which rotational speed was measured up to 1, 200°/s during quadrotor flips. In the meantime, gesture recognition is vital for in human-robot interaction. Hence, the neuromorphic gesture recognition system is urgently needed.

Ahn et al. (2011) were one of the first groups to use the DVS for gesture recognition when detecting and distinguishing between the 3 throws of the classical rock-paper-scissors game. It is noteworthy that their work was published in 2011, which predating the deep learning era. The DVS' inventors performed gesture recognition with spiking neural networks and leaky integrate-and-fire (LIF) neurons (Gerstner and Kistler, 2002; Lee et al., 2012a,b, 2014). Spiking neural networks (SNNs) are trainable models of the brain, thereby being suitable for neuromorphic sensors. In 2016 deep learning was first applied for gesture recognition with DVS (Park et al., 2016). With super-resolution technology by spatiotemporal demosaicing on the event stream, they trained a GoogLeNet CNN with the reconstructed information to classify these temporal-fusion frames and decode the network output with an LSTM. Amir et al. (2017) processed a live DVS event stream with IBM TrueNorth, a natively event-based processor containing 1 million spiking neurons. Configured as a convolutional neural network (CNN), the TrueNorth chip identifies the onset of a gesture with a latency of 105 ms while consuming <200 mW.

In fact, continuous gesture recognition is a task totally different from the segmented gesture recognition. For the segmented gesture recognition (Lee et al., 2012a; Amir et al., 2017), the scenario of the problem can be summarized as classifying a well-delineated sequence of video frames as one of a set of gesture types. This is in contrast with the continuous/online human gesture recognition where there are no a priori given boundaries of gesture execution. In a simple case where a video is segmented to contain only one execution of a human gesture, the system aims to correctly classify the video into its gesture category. In more general and complex cases, the continuous recognition of human gestures must be performed to detect the starting and ending times of all occurring gestures from an input video (Aggarwal and Ryoo, 2011). However, there has been no measurement till now for the detection performance in neuromorphic gesture recognition task. In brief, the continuous gesture recognition is the first step to reach online recognition though it is harder than the segmented gesture recognition (Wang et al., 2018).

However, the non-accumulated-image-based representation for event-driven recognition has not aroused enough attention. Both methods, Park et al. (2016) and Amir et al. (2017), belong to the semi-accumulated-frame-based representation and train CNN on the frames. Moreover, the CNN in Amir et al. (2017) was based on a neuromorphic hardware, which is not fully accessible to scientific and academic fields.There has been no pure deep network that can process the sequence of non-accumulated-frame-based representation for the gesture recognition task. A deep network should be urgently designed to process events or non-accumulated-frame-based representation sequence to explore a paradigm shift in neuromorphic vision community (Cadena et al., 2016). Because of the data nature of asynchronous, the direct raw event-based recognition might be unsatisfactory. How to learn a novel non-accumulated-frame-based representation for event-driven recognition therefore becomes a promising direction to reduce the noted negative effect and maximize the capability of the event-based sequence data.

The rest of this study is organized as follows: section 2 describes the preprocessing, the representation learning and RNN-HMM hybrid temporal classification for neuromorphic continuous gesture recognition. Section 3 verified the Neuro ConGD dataset collection, evaluation metrics and experimental results. Section 4 draws the conclusion of this study.

## 2. Methods

**Figure 1:**
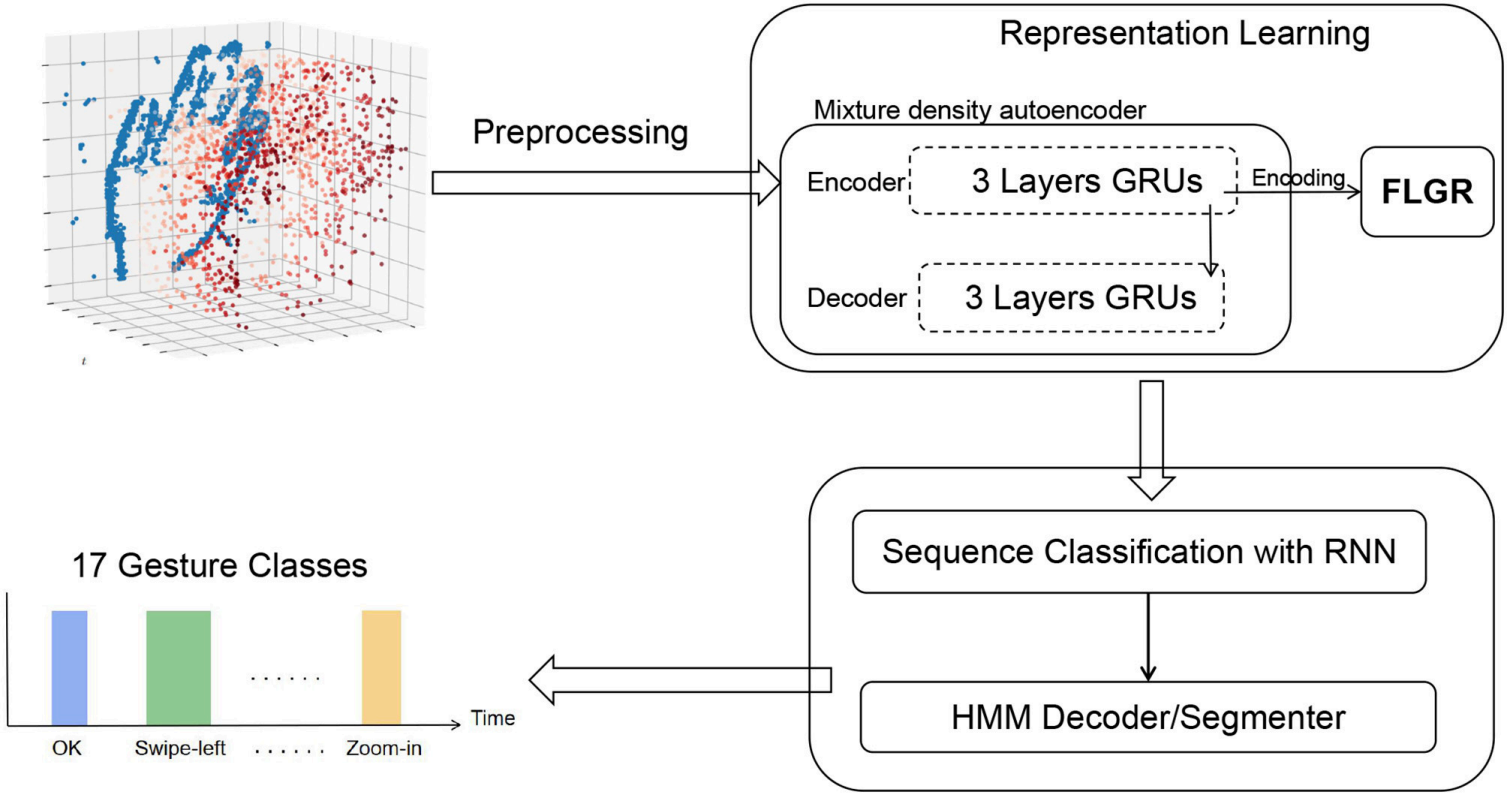
Overview of the framework for neuromorphic continuous gesture recognition. The autoencoder network was split into an encoder and a decoder, sharing information only along a single edge in the computational graph. The autoencoder was trained in an unsupervised way. The encoder part transform variable length events sequences of fixed duration into fixed-length vectors. The representation learning module learns a novel representation FLGR (Fixed Length Gists Representation). The FLGR sequences are then fed to the hybrid system with RNN and HMM to make a temporal classification.

In this section, the framework for neuromorphic continuous gesture recognition is to be described. The main idea of this study is shown in Figure 1.

The framework consists of two major parts, namely representation learning and temporal classification. In section 2.1, how the events triggered from DVS were preprocessed is first introduced. In section 2.2, a specific type of network *Mixture Density Autoencoder* is proposed to learn an efficient representation directly. In section 2.3, an RNN-HMM hybrid system is proposed to compute a label sequence from an input. The RNN provides localized classifications of each sequence element while the HMM segments the input based on the basis of the RNN output and deduces the most likely label sequence.

### 2.1. Event Preprocessing

The aim of preprocessing stage is to make the raw events data time-invariant, location-invariant and standardized. Each event was finally mapped from a 4-dimensional raw feature to a 6-dimensional preprepocessed feature in the end (see Equation 2).

To make the events sequence time invariant, a new variable δ*t* was introduced, which is defined as the time passed since the previous event, i.e., δ*t*^(*i*)^ = *t*^(*i*)^−*t*^(*i*−1)^ with a value of 0 as the base case. In such a way, arbitrary timestamp of the previous event was replaced.

To make the data location-invariant, we keep track of a mean μ_*x*_ with exponentially decaying weights, which gives more weight to recent events and can be cheaply computed in a streaming context such as online recognition. μ_*x*_ that tracks a quantity *x* through continuous time is defined as

(1) $$\begin{array}{l} \mu_{x}^{\left(i\right)}=\left(1-\alpha^{t^{\left(i\right)}-t^{\left(i-1\right)}}\right)\cdot x^{\left(i\right)}+\alpha^{t^{\left(i\right)}-t^{\left(i-1\right)}}\cdot \mu_{x}^{\left(i-1\right)} \end{array}$$

where *x*^(*i*)^ was observed at time *t*^(*i*)^. The parameter α controls how much weight is placed in past data.

We keep two means for each of *x* and *y*, one with λ = 1 s and another with λ = 50 ms. The first was supposed to track the main movement of the hand, while the second was to track fast movement like individual fingers.

In general, the preprocessing mapped each event from a 4-dimensional raw feature to a 6-dimensional feature as follows

(2) $$\begin{array}{l} \left(t^{\left(i\right)},x^{\left(i\right)},y^{\left(i\right)},p^{\left(i\right)}\right)\mapsto \left(\delta t^{\left(i\right)},\delta x_{\lambda =1\;\text{s}}^{\left(i\right)},\delta x_{\lambda =50\;\text{ms}}^{\left(i\right)},\delta y_{\lambda =1\;\text{s}}^{\left(i\right)},\delta y_{\lambda =50\;\text{ms}}^{\left(i\right)},p^{\left(i\right)}\right) \end{array}$$

where $\delta x_{\lambda =1\;\text{s}}^{\left(i\right)}=x^{\left(i\right)}-\;\mu_{x,\lambda =1\;\text{s}}^{\left(i\right)}$, $\delta x_{\lambda =50\;\text{ms}}^{\left(i\right)}=x^{\left(i\right)}-\mu_{x,\lambda =50\;\text{ms}}^{\left(i\right)}$,$\delta y_{\lambda =1\;\text{s}}^{\left(i\right)}=y^{\left(i\right)}-\mu_{y,\lambda =1\;\text{s}}^{\left(i\right)}$, $\delta y_{\lambda =50\;\text{ms}}^{\left(i\right)}=y^{\left(i\right)}-\mu_{y,\lambda =50\;\text{ms}}^{\left(i\right)}$.

### 2.2. Representation Learning for FLGR

The aim of the representation learning stage focused on learning feature from the variable length events sequence. A mixture density network following the autoencoder architecture proposed in Cho et al. (2014) was utilized, which was originally employed for machine translation. Both the encoder and decoder of mixture density autoencoder consist of Gated Recurrent Units (GRU). The representations learned by the autoencoder is termed as *Fixed Length Gist Representation (FLGR)*. First, FLGR encode the gist of the input. Second, the variable length event sequences of fixed duration are transformed into fixed-length vector with the representation learning. We hope to inspire greater efforts along the lines of the non-accumulated-image-based representation research on neuromorphic vision.

#### 2.2.1. Mixture Density Autoencoder

The aim of the mixture density autoencoder is to learn a low-dimensional representation of the input data from which it can later reconstruct the input. Graves (2013) proposed mixture density network to generate handwriting sequence from a trained network by learning input sequence's distribution. The property of mixture density network was exploited to make the autoencoder transform variable length event sequences of fixed duration into fixed-length vectors.

**Figure 2:**
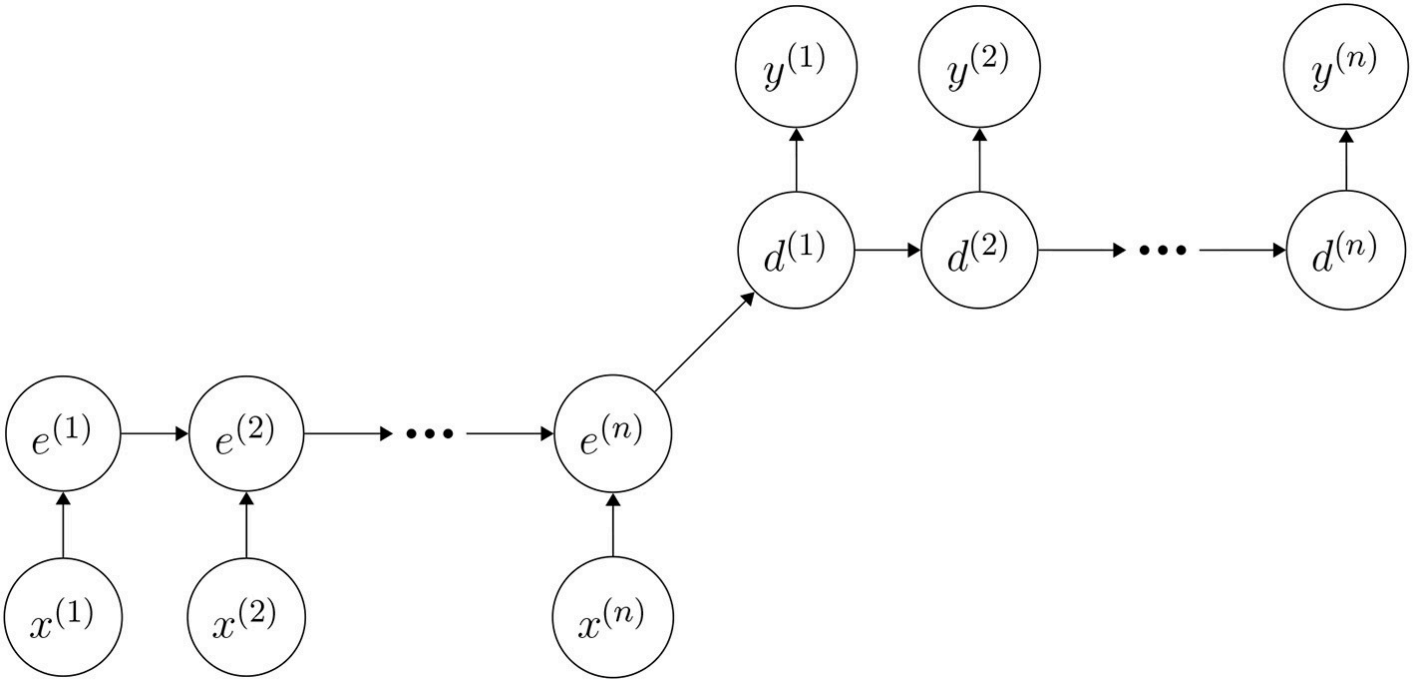
Sketch of our autoencoder architecture that encodes an input sequence *x* of length *n* into hidden states *e*. The decoder is trained to decode the last hidden state *e*^(*n*)^ into a sequence *y*^(1)^, …, *y*^(*n*)^ resembling the input. Each *y*^(*i*)^ is a non-negative vector whose entries sum up to 1 and its *j*-th entry encodes the networks belief that the *j*-th word should be placed at this point in the output sequence. Note that this is a sketch for intuitive understandability. Both encoder and decoder have 3 layers GRUs separately. Implementation details can be seen in section 2.4.

The autoencoder network was split into an encoder and a decoder, sharing information only along a single edge in the computational graph (see Figure 2). This edge initializes the decoders hidden state with the final hidden state of the encoder. It is the figurative funnel in the network as it has to encode the complete input sequence. The mixture density autoencoder was trained to produce a probability distribution over sequences rather than sequences directly. Our network processed an input sequence of length *n*, where *n* is variable, by encoding the complete sequence first. Subsequently, it used the decoder to produce a distribution over a sequence of length *n* and computing a loss between the two sequences for training. The mixture density networks output parameterizes a distribution, which is a mixture of Gaussians over the real attribute and a Bernoulli distribution over the categorical attribute. It is noteworthy that the outputs of our mixture density autoencoder are parameters of mixture distribution which are corresponding to the input events sequence. These parameters were used to reconstruct the sequence. During training we use encoder together as an autoencoder for the sequence and derive the training signal from the reconstruction error of the sequence. Then, we throw away the decoder and rely solely on the encoder to generate enriched, learned FLGR representation.

#### 2.2.2. Gated Recurrent Unit

A crucial property for our recurrent model refers to the ability to operate over input events sequence. In the proposed mixture density autoencoder, both encoder and decoder consist of 3 layers GRUs. Though works for sequence encoding and classification often leverage Long Short-Term Memory (LSTM) cells, it was reported that a GRU-based architecture exhibiting slightly better performance is more robust over a wider range of hyperparameters and has fewer parameters, suggesting the slightly faster training and better test-time performance. This is consistent with empirical findings from prior work on deep recurrent models in other domains (Jozefowicz et al., 2015). GRU merged the cell state into the hidden state *h*^(*t*)^, combined the input and forget gates into a single update gate *z*^(*t*)^ and replaced the output gate with a reset gate *r*^(*t*)^ with no equivalent in LSTM.

Thus, at each time step *t*, we took the hidden state *h*^(*t*)^ of the final GRU layer in the recurrent stage as our sequence encoding, where *h*^(*t*)^ is defined as:

(3) $$r^{\left(t\right)}=\sigma \left(W_{r}x^{\left(t\right)}+U_{r}h^{\left(t-1\right)}\right)$$

(4) $$z^{\left(t\right)}=\sigma \left(W_{z}x^{\left(t\right)}+U_{z}h^{\left(t-1\right)}\right)$$

(5) $${\tilde{h}}^{\left(t\right)}=\tanh\left(Wx^{\left(t\right)}+U\left(r^{\left(t\right)}◦h^{\left(t-1\right)}\right)\right).$$

(6) $$h^{\left(t\right)}=z^{\left(t\right)}◦h^{\left(t-1\right)}+\left(1-z^{\left(t\right)}\right)◦{\tilde{h}}^{\left(t\right)}$$

### 2.3. RNN-HMM Hybrid for Temporal Classification

The aim of temporal classification was to transform an event sequence to a sequence of 17 gesture labels. Wu et al. (2018) trained an event-driven RNN on DVS-MNIST dataset, verifying the capability of RNN to process the event-based classification task. RNNs consisting of LSTM units or GRU units are efficient methods for continuous gesture recognition (Chai et al., 2016; Cui et al., 2017). Moreover, the hybrid system combined with neural network and hidden Markov model (HMM) will significantly enhance the performance of temporal classification (Abdel-Hamid et al., 2012; Gaikwad, 2012). Based on the above information, an RNN-HMM hybrid for temporal neuromorphic continuous gesture classification was developed. Our RNN-HMM hybrid consists two modules: sequence classification with RNN to produce a distribution of labels and HMM to decode the distribution of labels into correct gesture label. Though this study focus on the case of gesture recognition, we hope to inspire more efforts on neuromorphic temporal classification tasks based on the proposed RNN-HMM hybrid.

#### 2.3.1. Sequence Classification With Recurrent Neural Network

The aim of sequence classification was to take an input sequence and produce a distribution of labels. A RNN was employed for event sequence classification. To classify a continuous gesture sequence, localized classifications were required to be decoded into global classifications. In other words, the network should assign each unit of input FLGR sequence to one of 17 classes. The 17 classes contain 16 gestures plus the *blank* label (see section 3.1 for the definition of gesture classes).

**Figure 3:**
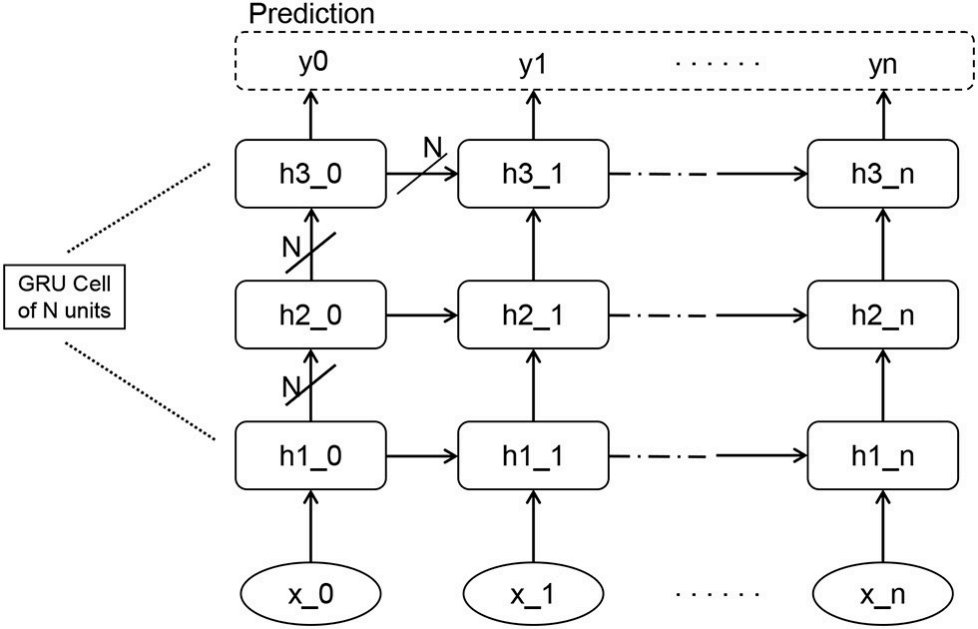
The RNN network consists of three layers of GRUs, each of which has N units, i.e., 256 units in our setting. The output of RNN is transformed with Softmax to parameterize a distribution over the classes. Finally the network can be trained to produce prediction y.

Figure 3 shows the structure of our RNN network. The RNN consists of three GRU layers, two fully-connected layers with *tahn* activation and finally a fully-connected layer that projects the output down into ℝ^17^. The definition of GRU and the reason to choose GRU instead of other recurrent units like LSTM are explained in section 2.2.2. In our RNN sequence classifier, the learning rate, decay rate, and neuron number of each GRU were set to 10^−3^, 0.95, and 256, respectively. The loss function is cross entropy measuring the difference between the labels and predicted outputs. The output is transformed with SoftMax to parameterize a multinoulli distribution over the output classes.

**Figure 4:**
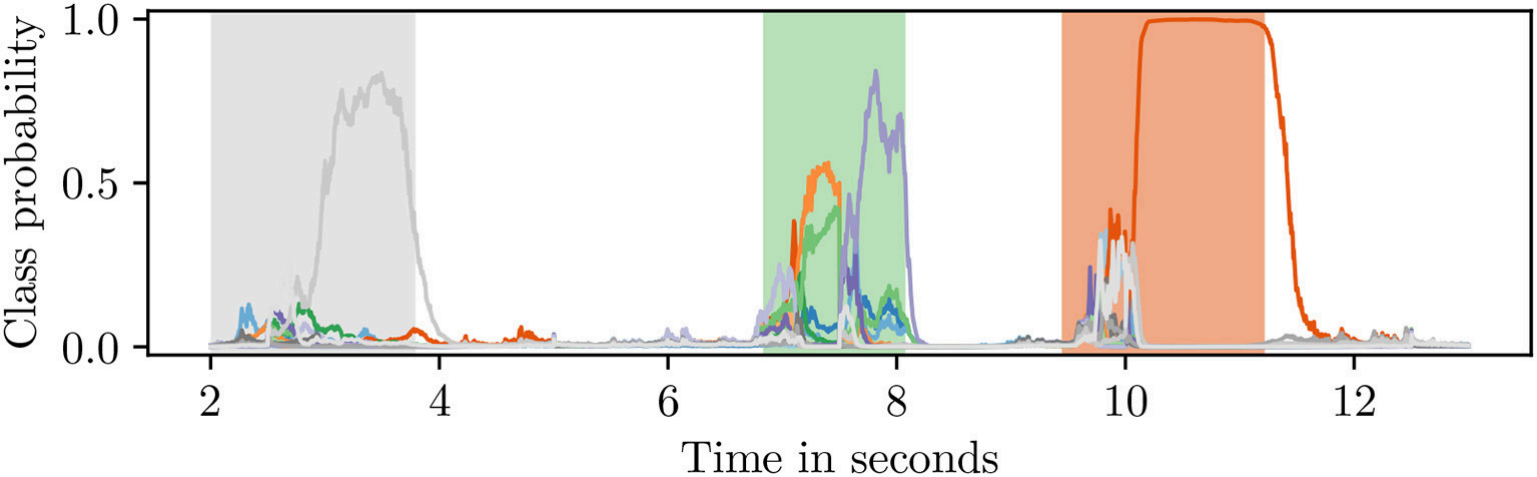
Class probabilities attained from an RNN sequence classifier. The shaded regions in the background designate the ground truth (best viewed in color).

The output of a sequence classifier is shown in Figure 4. Since the *blank* class taked up nearly 50% of the training data, the classifier recognized non-gesture data with high accuracy. When an activity was detected, the classifier assigned high probabilities to multiple classes at first until it discerned a single label as the correct one.

#### 2.3.2. HMM Decoding and Segmentation

**Figure 5:**
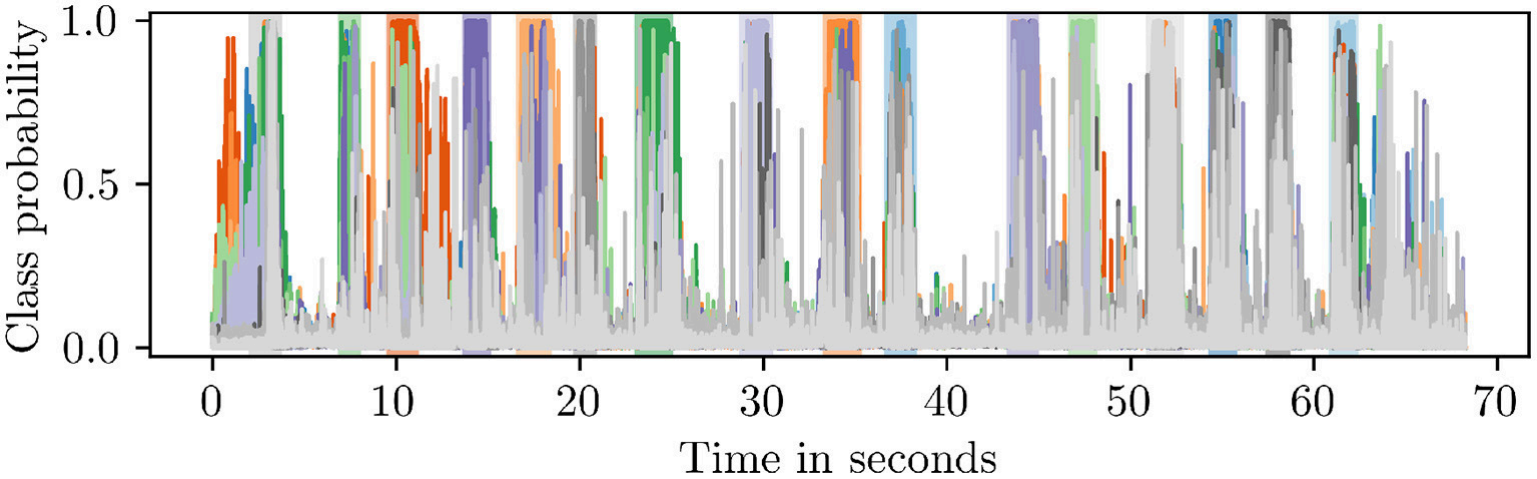
Noisy classifications produced by an RNN classifier (best viewed in color).

The major goal of our HMM is to process the noisy classification produced by sequence classifier. Sequence classifier with RNN points out which gesture most likely happens at each point in time. However, there exist a huge amount of noisy classifications produced by the RNN sequence classifier (See Figure 5). For instance, a *swipe-down* gesture might be classified as *rotate-outward* for the first few milliseconds, then *swipe-up* for another few and finally as *swipe-down* for the rest of the activity. Furthermore, this sequence of probability distributions should be deciphered into a single *swipe-down* label. The solution was reached using HMMs decoding (HMM decoder).

A HMM consists of a Markov chain of hidden states *z*^(*t*)^ ∈ {1, …, *K*}, an observation model *x*^(*t*)^ and a transition model expressed as transition matrix *A*. An HMM models a situation where a state of interest is only indirectly observable through emissions at each timestep. We have a sequence of local classifications of each frame into of 17 classes and would like to derive the true underlying sequence of gestures. An HMM helps us incorporate the knowledge that a state *i* might be observed as any other state for a short while through the observation matrix B. An efficient algorithm *Viterbi decoding* was employed to decode an observation sequence into the most likely underlying state sequence is. *Viterbi decoding* produces the most likely sequence of hidden states

(7) $$\begin{array}{ll} z^{\left(1\right)},\ldots ,z^{\left(n\right)} & =\underset{z^{\left(1\right)},\ldots ,z^{\left(n\right)}}{\text{arg max}}p\left(z^{\left(1\right)},\ldots ,z^{\left(n\right)}|x^{\left(1\right)},\ldots ,x^{\left(n\right)}\right) \end{array}$$

(8) $$=\underset{z^{\left(1\right)},\ldots ,z^{\left(n\right)}}{\text{arg max}}p(z^{\left(1\right)})\cdot \prod_{t=2}^{n}p(z^{\left(t\right)}|z^{\left(t-1\right)})\cdot \prod_{t=1}^{n}p(x^{\left(t\right)}|z^{\left(t\right)})$$

(9) $$\begin{array}{l} =\underset{z^{\left(1\right)},\ldots ,z^{\left(n\right)}}{\text{arg max }}\text{log }\pi_{z^{\left(1\right)}}+\sum_{t=2}^{n}\text{log }A_{z^{\left(t-1\right)},z^{\left(t\right)}}\\ +\sum_{t=1}^{n}\text{log }p(x^{\left(t\right)}|z^{\left(t\right)}) \end{array}$$

given a sequence of observations. Since the RNN classifier with softmax layer produces $p(z^{\left(t\right)}|x^{\left(t\right)})$ instead of $p(x^{\left(t\right)}|z^{\left(t\right)})$, the decoding objective can be rewritten in accordance with Bayes' theorem.

(10) $$\begin{array}{l} =\underset{z^{\left(1\right)},\ldots ,z^{\left(n\right)}}{\text{arg max}}\text{log }\pi_{z^{\left(1\right)}}+\sum_{t=2}^{n}\text{log }A_{z^{\left(t-1\right)},z^{\left(t\right)}}\\ +\sum_{t=1}^{n}\left(\text{log }p(z^{(t}|x^{\left(t\right)})+\text{log }p(x^{\left(t\right)})-\text{log }p(z^{\left(t\right)})\right) \end{array}$$

The $p(x^{\left(t\right)})$ term is irrelevant to the Argmax asit does not depend on *z*.

(11) $$\begin{array}{l} =\underset{z^{\left(1\right)},\ldots ,z^{\left(n\right)}}{\text{arg max}}\text{log }\pi_{z^{\left(1\right)}}+\sum_{t=2}^{n}\text{log }A_{z^{\left(t-1\right)},z^{\left(t\right)}}\\ +\sum_{t=1}^{n}\left(\text{log }p(z^{(t}|x^{\left(t\right)})-\text{log }p(z^{\left(t\right)})\right) \end{array}$$

(12) $$\begin{array}{l} =\underset{z^{\left(1\right)},\ldots ,z^{\left(n\right)}}{\text{arg max}}\text{log }\pi_{z^{\left(1\right)}}+\sum_{t=2}^{n}\text{log }A_{z^{\left(t-1\right)},z^{\left(t\right)}}\\ +\sum_{t=1}^{n}\text{log }p(z^{(t}|x^{\left(t\right)}) \end{array}$$

The Viterbi algorithm finds the maximizer by computing the probability of being in state *j* at time *t* since the most probable path is taken.

(13) $$\delta_{t}(j)=\underset{z^{\left(1\right)},\ldots ,z^{\left(t-1\right)}}{\max}p(z^{\left(1\right)},\ldots ,z^{\left(t-1\right)},z^{\left(t\right)}=j|x^{\left(1\right)},\ldots ,x^{\left(t\right)})$$

The key insight here is that the most probable path to state *j* at time *t* must be the one that maximizes the joint probability of being in state *k* at time *t* − 1 and transitioning from *k* to *j*, i.e.,

(14) $$\delta_{t}(j)=\underset{i}{\text{max }}\delta_{t-1}(i)\;\cdot \;A_{ij}\;\cdot \;B_{j,x^{\left(t\right)}}$$

If computing δ for *t* from 1 to *n* and store the maximizer *i* in another table α_*tj*_, you can find the most probable final state as $z^{\left(n\right)}={\text{arg max}}_{i}\delta_{n}\left(i\right)$ and work your way back to *t* = 1 by following the $\alpha_{n,z^{\left(n\right)}}$ to the predecessor state and so forth. This explanation is summarized from Murphy (2012).

Since the the observation matrix *B* can be derived from the output of RNN classifier with SoftMax layer, the constructing process of an HMM decoder was reduced to find *A* and π.

**Figure 6:**
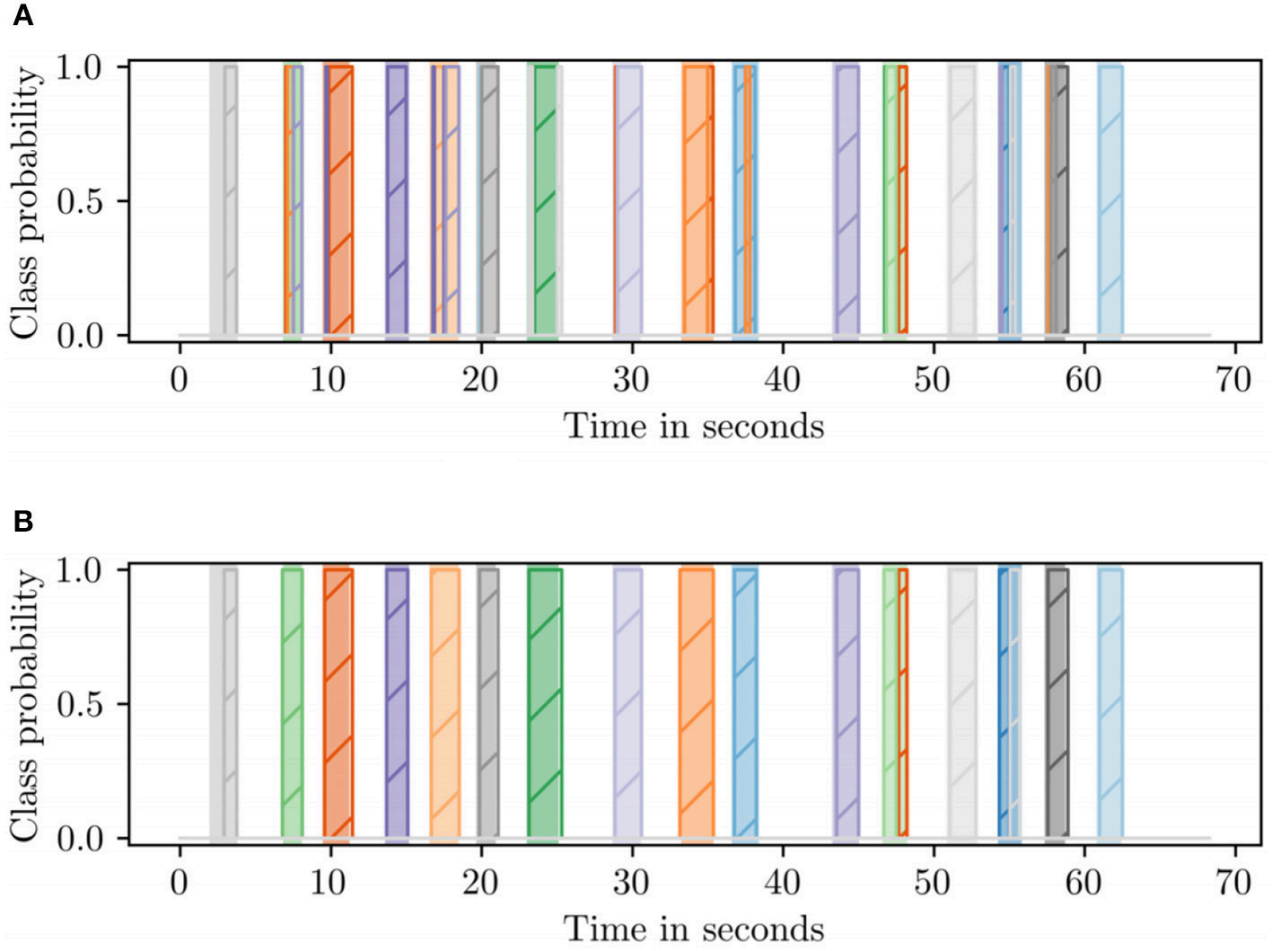
Comparison of the two decoding methods on the same recording. The shaded regions in the background denote the ground truth (best viewed in color). **(A)** Direct HMM decorder. **(B)** HMM segmenter plus segment decoding.

Figure 6A shows that an HMM decoder is capable of recognizing the points of activity in a sequence of local classifications, and it is also reasonably accurate in decoding them into the true label.

However, there were numerous spurious labels mixed in with the true labels (see Figure 6A). To solve the mixing problem, an HMM segmenter was developed, and the decoding process was devided into two parts, first, an HMM with just two states, *gesture* and *blank*, segments the sequence; subsequently, a second HMM produces a single label for each segment.

The HMM segmenter was constructed in the same way as the decoder with the twist that all gestures are combined into a single hidden state *gesture*. When the HMM segments a recording, the probability of the *gesture* state is the sum of all gesture probabilities. To suppress the remaining spurious activations, all segments shorter than 500 ms were also filtered out since we know from the dataset statistics that the shortest gesture is over a second long on average according to dataset statistic. Figure 6B shows the contamination of mixing labels was almost gone compared with the result in Figure 6A. Thus, the results were improved after implementing HMM segmenter.

### 2.4. Network Training and Implementation

The aim of the training process is to estimate the model parameters in our architecture. During training we used encoder and decoder together as a mixture density autoencoder. We reconstructed the sequence by means of mixture distribution produced by autoencoder. We derive the training signal from the reconstruction error of the sequence. The encoder part of trained encoder-decoder was employed to generate FLGR data from variable events sequence. Given the sequence of FLGR, the hybrid system with RNN classifier and HMM was trained to predict the corresponding label.

The training events segment and batch were generated as follows: the time window *T*_*w*_ with a fixed duration was constructed as a segment. Events fell into different *T*_*w*_ with variable length *L*_*i*_. The max value of *L*_*i*_ among different *T*_*w*_ was then computed as *L*_*max*_. Each batch contains several *T*_*w*_ with the amount of batch size *S*_*batch*_. The final data of a batch has the shape of (*S*_*batch*_, *L*_*max*_, *S*_*event*_) where *S*_*event*_ denotes the feature size in each processed events. In our training, the *T*_*w*_, *S*_*batch*_, and *S*_*event*_ were set to 2.5 ms, 32 and 6, respectively.

In our implementation, the training procedure of our mixture density autoencoder is as follows. Both the encoder and decoder are 3 layers of GRUs with 256 neurons of each. The encoder receives preprocessed events in ℝ^6^. The decoder produces parameters for a 10-component mixture distribution of Gaussians with diagonal covariance matrices over ℝ^5^ and a single parameter for a Bernoulli distribution over {−1, 1}. This adds up to 10 component weights, 10·5 = 50 mean parameters, 10·5 = 50 diagonal convariance matrix entries and a single Bernoulli parameter, in total of 111 parameters. To project its 256-dimensional output into the ℝ^111^, the decoder has a single fully-connected layer with weight matrix and bias term but without non-linearity on top. According to the output distribution, the loss is the negative log-likelihood of the input sequence. The network weights are learned using the mini-batch gradient decent with batch size 32. The optimizer is Adam (Kingma and Ba, 2015) with a learning rate of 10^−4^ and an exponential decay rate of 0.95 to the power of the current epoch. The gradients were clipped at a norm of 5. This also helps to solve numerical instabilities if the covariances of the mixture distribution become really small.

For the RNN sequence classifier, the learning rate, decay rate, and neuron number of each GRU were set to 10^−3^, 0.95, and 256, respectively. The loss function was cross entropy measuring the difference between the labels and predicted outputs.

The construction of an HMM decoder from the training data aimed to find *A* and π. We define the *A*_*i*, 17_ entries, the transition probability from gesture *i* to *blank*, as the proportion of frames belonging to class *i* that transition to *blank* and *A*_*i, i*_, the self-transition probability, as 1 − *Ai*, 17. The transition probability from *blank* to any of the gestures was the proportion of gesture gists following blank gists, and the self-transition probability acted as the complementary part.

For the programming platform, a Titan X graphics card and an Intel Core i7-5930K processor were utilized for training, processing, and implementation.

## 3. Experiments

In this section, the Neuromorphic Continuous Gesture Dataset (Neuro ConGD) and the evaluation protocol are to be described. The dataset contains the raw recorded events and the preprocessed data. The experimental results of the proposed method on this dataset were reported and compared with the baselines.

### 3.1. Neuro ConGD Dataset

Numerous gesture datasets have been created in recent years, as thoroughly review in Ruffieux et al. (2014). Most of the datasets were recorded with frame-based camera, e.g., the conventional color camera, the stereo camera and the Kinect. Hu et al. (2016) reported the urgent need for neuromorphic dataset for further research in the event-based computer vision. One of the contributions of this study is that a new neuromorphic continuous gesture (Neuro ConGD) dataset was collected with an event-based camera.

**Figure 7:**
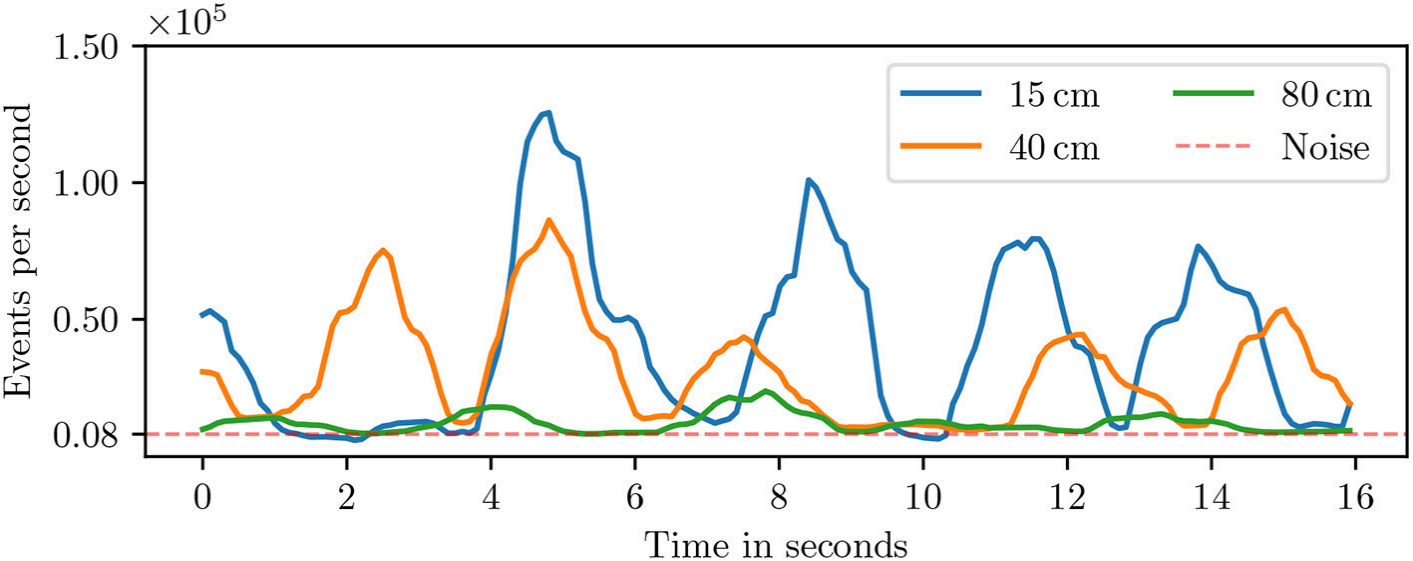
Event density for various distances between the hand and the DVS.

The Neuro ConGD dataset was recorded with a DVS sensor which has a spatial resolution of 128x128 pixels. An appropriate distance between hand and DVS should be first selected to make gesture distinguishable from noise. Figure 7 shows the event rates of three recordings taken at three different distances between hand and DVS. A noise event rate of nearly 8 keps was measured when the DVS was directed toward a static scene which is the baseline rate between gestures regardless of the distance. The peaks in the event rate show that the event rate above baseline is proportional to the distance between hand and DVS. However, small distance makes the hand gesture leave the DVS' field of view while recordings with a distance of over 80 cm are almost indistinguishable from noise. Accordingly, the distance was kept from 40 to 50 cm.

**Figure 8:**
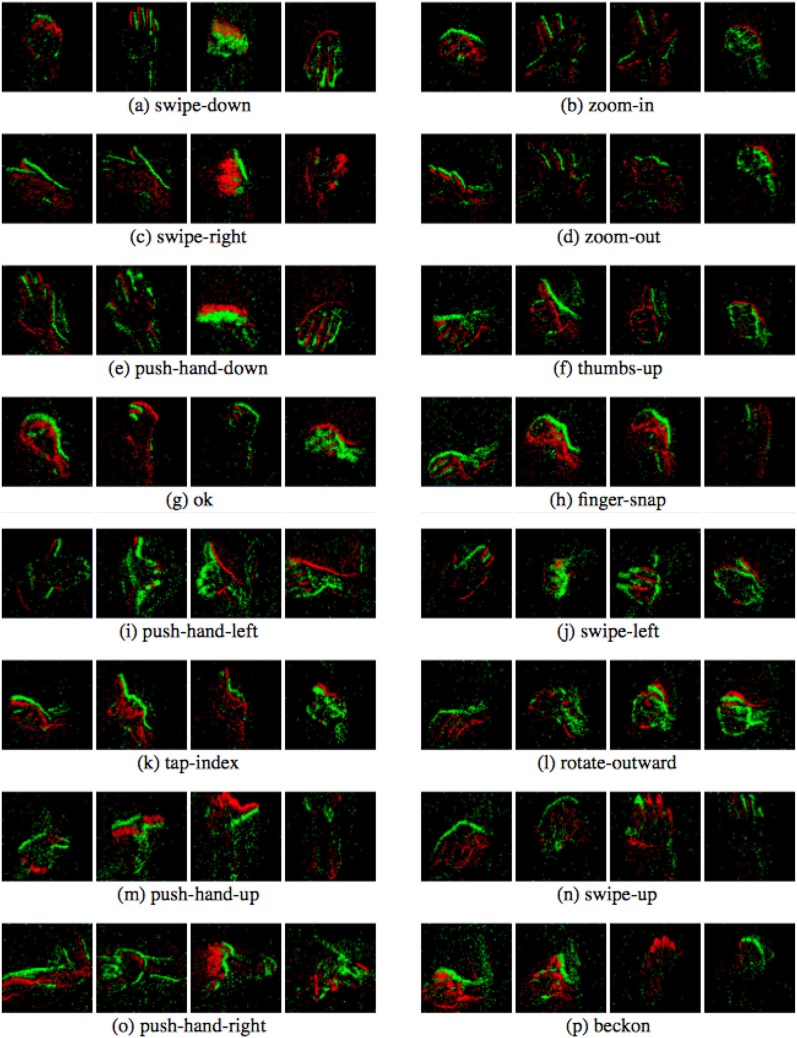
A dataset overview demonstrating the 16 gestures, each of which contains 4 sub-figures of different timestamps (best viewed in color).

Sixteen gesture classes were defined with an additional class *blank*, as listed in Table 1. Neuro ConGD dataset comprises 2,040 instances of a set of 17 gestures recorded in random order. The Neuro ConGD dataset was split into 3 mutually exclusive subsets, namely the training, the validation and the testing set. The training set was performed by 4 subjects. The validation set was performed by 2 subjects. The testing set was also performed by 2 subjects. The gestures include beckoning, finger-snap, ok, push-hand (down, left, right, up), rotate-outward, swipe (left, right, up), tap-index, thumbs-up, zoom (in, out) (See Figure 8).

**Table 1:** Information of the Neuro ConGD dataset.

**Set**	**No. of labels**	**No. of gestures**	**No. of sequences**	**No. of subjects**	**Preprocessing provided**	**Labels provided**
Training	17	1,360	80	4	Yes	Yes
Validation	17	340	20	2	Yes	Yes
Testing	17	340	20	2	Yes	No

A purpose-built labeling software was developed, and each recording was manually annotated by labeling a list of start and end timestamps for each gesture with the name of gesture class.

### 3.2. Evaluation Metrics

#### 3.2.1. Mean Jaccard Index for Overall Recognition Performance

The Jaccard index is to measure the average relative overlap between the actual and the predicted sequences of timestamps for a given gesture (Pigou et al., 2018; Wang et al., 2018). For a sequence *s*, let *G*_*s, i*_and *P*_*s, i*_ be binary indicator vectors for which 1-values correspond to timestamps in which the *i*^*th*^ gesture/action label is being performed. For the sequence s, The Jaccard Index for the *i*^*th*^ class is defined as:

$$\begin{array}{l} J_{s,i}=\frac{G_{s,i}\cap P_{s,i}}{G_{s,i}\cup P_{s,i}} \end{array}$$

where *G*_*s, i*_ denoted the ground truth of the *i*^*th*^ gesture label in sequence *s*, and *P*_*s, i*_ is the prediction for the *i*^*th*^ label in sequence *s*. Subsequently, for the sequence *s* with *l*_*s*_ true labels, the Jaccard Index *J*_*s*_ is calculated by:

(16) $$\begin{array}{c} J_{s}=\frac{1}{l_{s}}\sum_{i=1}^{L}J_{s,i} \end{array}$$

For all test sequences *S* = *s*_1_, …, *s*_*n*_ with 17 gestures, the mean Jaccard Index $\bar{J_{S}}$ serves as the evaluation criteria, and it is calculated by:

(17) $$\bar{J_{S}}=\frac{1}{n}\sum_{j=1}^{n}J_{s_{j}}$$

#### 3.2.2. F-Score for Detection Performance

One difficulty of continuous gesture recognition is to detect the start time point and end time point of a gesture. For the segmented gesture recognition, the scenario of the problem can be summarized as classifying a well-delineated sequence of video frames as one of a set of gesture types. This contrasts with continuous human gesture recognition where there is no priori given boundary of gesture execution. This requires the system to distinguish the blank and non-blank (gestures) class in each time point. To assess the detection performance, we keep the *blank* class and merge the rest 16 gestures be one class as *Ges*. Then, the task now is to detect non-blank gestures without recognizing the specific kind of class. In the prediction and ground truth, the value of *blank* and *Ges* are 0 and 1, respectively. Subsequently, the *F-score* measure (Sokolova and Lapalme, 2009) is defined as:

(18) $$F_{\text{ score }}=2*\frac{\text{ Precision }*\text{ Recall }}{\text{ Precision }+\text{Recall}}$$

### 3.3. Experimental Results

To illustrate the effectiveness of FLGR representation, a baseline where the RNN sequence classifier are trained with variable length events sequences was designed. The proposed frameworks with protocol of mean Jaccard Index $\bar{J_{S}}$ and *Fscore* were assessed, and they were compared with baseline.

Table 2 shows the final results across combinations of input representation and decoding method. An RNN baseline with inputs of event sequences was designed. The case of baseline achieved 63.3 % $\bar{J_{S}}$ accuracy, which is reasonable and acceptable but still challenging. The case of baseline verified the fundamental capability of our RNN network in event-driven recognition. Our architecture was improved based on FLGR representation and late HMM decoding. After FLGR representation learning, the $\bar{J_{S}}$ accuracy was improved by more than 15%. The *Fscore* for detection result was improved to 94.4%. The best result was achieved on FLGR representaton learning with an RNN classifier and decoding method with an extra segmentation step. The averages of the best $\bar{J_{S}}$ and *Fscore* were up to 86.9 and 96.6%, respectively. For the cases among FLGR, the $\bar{J_{S}}$ accuracy is also improved by more than 8% after applying HMM segmenter. Table 2 shows the large improvement after applying FLGR representation, which verifies the enhanced efficiency of FLGR representation for training a sequence classifier.

**Table 2:** Performance measured on the testing dataset with mean Jaccard Index $\bar{J_{S}}$ and *Fscore*.

**Methods**	**$\bar{J_{S}}$**	***Fscore***
Events+RNN (baseline)	0.633	0.873
FLGR+RNN	0.788	0.944
FLGR+RNN+Hmm	0.817	0.963
**FLGR+RNN+HmmSeg**	**0.869**	**0.966**

*$\bar{J_{S}}$ was measured for overall performance of recognizing 17 gestures. Fscore was measured for detection performance. The higher the value is, the better the method will perform. Events, Preprocessed events; RNN, Recurrent neural network for sequence classification; Hmm, HMM decoder; HmmSeg, HMM segmenter. The bold values mean the best results achieved among the listed methods*.

## 4. Conclusion and Discussion

In this study, a neuromorphic continuous gesture recognition system was proposed, and how it can benefit from FLGR representation learning and RNN-HMM hybrid was analyzed. A novel representation learning method was presented to learn non-accumulated-frame-based FLGR representation from DVS events streams. An RNN-HMM hybrid was proposed for the event-based sequence classification. A new labeled neuromorphic continuous gesture dataset Neuro ConGD was created with more than 2,040 instances of 17 gesture classes from 120 events sequences. An RNN classifier was developed as baseline, and the architecture with another 3 different paths on our dataset was improved. According to the experimental results, we could achieve an $\bar{J_{S}}$ of 86.9% for recognition performance and an average *Fscore* of 96.6% for detection performance, with a mixture density autoencoder for FLGR representation learning, a RNN for sequence classification and an HMM segmentation process.

Compared with the conventional accumulated-frame-based representation of DVS events streams, FLGR marks two major contributions: First, FLGR is a sequence learned from mixture density autoencoder and preserve the nature of event-based data better. Second, FLGR has a data format of fixed length, and it is easy to feed to sequence classifier. With a preliminary result in this work, we believe that our FLGR representation learning and RNN-HMM hybrid is believed to have large potential to be transferred to neuromorphic vision in other pattern recognition and sequence classification tasks. We hope to inspire the research on the event-based sequence classification tasks with the non-accumulated-frame-based representation.

There are still several ways the recognition performance of this system can be improved. One idea would be to increase the information content of the learned representations at times of low event density. Then, the autoencoders state was reset to zero between each time window. This can be improved by using the autoencoder in a rolling fashion by not resetting the hidden states between time windows. This could help to classify stretches of time in gestures of low activity, e.g., the turning point of a swiping gesture. Another idea would be to use a bidirectional neural network so that the subsequent fully-connected layers can take past as well as future context into account and avoid the phase of confusion at the beginning of a gesture. that can incorporate requirements like a minimum length of a hidden state directly into the model instead of having to post-process the decoding and segmentations.

## Author Contributions

GC, JNC, and ML contributed equally to this work. GC, JNC, ML, JC, FR, and AK did conception and design, analysis and interpretation of data, drafting and revising the article. ML and GC performed the experiments and acquired the data. JC provided the Dynamic Vision Sensor.

### Conflict of Interest Statement

The authors declare that the research was conducted in the absence of any commercial or financial relationships that could be construed as a potential conflict of interest.
